# ^18^F-FDG PET/CT of advanced gastric carcinoma and association of HER2 expression with standardized uptake value

**Published:** 2014

**Authors:** Jin Suk Kim, Shin Young Park

**Affiliations:** 1 Department of Nuclear Medicine, Konyang University College of Medicine, Daejeon, Korea; 2 Department of Pathology, Konyang University College of Medicine, Daejeon, Korea; 3 Konyang University Myunggok Medical Research Institute, Daejeon, Korea

**Keywords:** Advanced gastric carcinoma, HER2, PET-CT

## Abstract

**Objective(s):**

Expression of HER2 in gastric carcinoma has direct prognostic and therapeutic implications in patient management. The aim of this study is to determine whether a relationship exists between standardized uptake value (SUV) and expression of HER2 in advanced gastric carcinoma.

**Methods:**

We analyzed the ^18^F-FDG PET/CT results of 109 patients that underwent gastrectomy for advanced gastric carcinoma. The ^18^F-FDG PET/CT imaging was requested at the initial staging before surgery. The examinations were evaluated semi-quantitatively, with calculation of maximum standardized uptake values (SUV_max_). The clinicopathologic factors, including HER2 overexpression, were determined from tissue obtained from the primary tumor. Metabolic and clincopathologic parameters were correlated using a *t*-test, one way ANOVA and chi-square test.

**Results:**

Immunohistochemically, 26 patients (23.8%) showed HER2 overexpression. This overexpression was significantly associated with high SUV level (*P*=0.02). The SUV level was significantly correlated with tumor size (*P*=0.02) and differentiation (*P*<0.001), and Lauren histologic type (*P*=0.04). Multivariate analysis showed HER2 overexpression, large tumor size, and differentiation (*P*=0.022, *P*=0.002, *P*<0.001) were significantly correlated with the high level of SUV in advanced gastric carcinoma. No association was found between SUV and T stage and lymph node metastasis. A receiver-operating characteristic curve demonstrated a SUV_max_ of 3.5 to be the optimal cutoff for predicting HER2 overexpression (sensitivity; 76.9%, specificity; 60.2%).

**Conclusion:**

An association exists between high SUV and HER2 overexpression and ^18^F-FDG PET/CT could be a useful tool to predict the biological characteristics of gastric carcinoma.

## Introduction

Gastric carcinoma is the fourth most common cancer in the world, and it is the second most common cause of death from cancer (1). Early detection of this cancer is increasing due to diagnosis technology advancements and increased frequency of checkups. The 5-year survival rate for gastric cancer has been showing an increasing trend (2). However, advanced gastric carcinoma (AGC) is still frequently detected, and prognosis of AGC is not promising despite current treatments that include curative gastrectomy and combination chemotherapy (2).

The gene for human epidermal growth factor receptor 2 (HER2) protein (also known as c-erbB2) is a transmembrane tyrosine kinase receptor and a member of the epidermal growth factor receptor family (3). overexpression of HER2 has been found to promote tumorigenesis and to be involved in the pathogenesis of several human cancers (4). Overexpression of HER2 and amplification of the HER2 gene in breast carcinoma has been associated with poor prognosis (5). However, HER2 status has become an important predictive biomarker for treatment with HER2 inhibitors such as trastuzumab (Herceptin^®^, Roche) and the tyrosine kinase inhibitor lpaptinib (Tyberb^®^/Tykerb^®^, GSK) (6).

Amplification and overexpression of HER2 have been reported in malignancies other than breast carcinoma, such as colon, prostate, lung and gastric carcinomas (7-9). Trastuzumab has recently been shown to improve the prognosis of HER2-positive AGC (10). Combination chemotherapy, that consists 5-fluorouracil (5-FU) or capecitabine plus cisplatin with trastuzumab, has shown a significantly higher response rate and resulted in a longer survival than chemotherapy alone (10). HER2 has shown to be predictive for the treatment with trastuzumab in AGC as well as breast carcinoma (10).

PET imaging with ^18^F-fluorodeoxyglucose (FDG) is an important noninvasive technique that is frequently applied in the diagnosis and staging of different type of cancer. The degree of ^18^F- FDG uptake in lung carcinoma, lymphoma, and esophageal carcinoma also correlates well with validated prognostic markers (11). In gastric carcinoma, however, attempts to correlate ^18^F-FDG uptake with known prognostic markers have been disturbed by technical limitations, various histological types, and normal physiologic FDG uptake.

In the present study, we investigated the relationship between SUV_max_ in AGC and clinicopathologic factors including HER2 status.

## Methods

### Patients

Between August 2007 and December 2011, 109 patients underwent gastrectomy for AGC and were studied with ^18^F-FDG PET/CT before surgery. None of these patients was treated with preoperative chemotherapy or radiation therapy.

For the purpose of this study, the following characteristics were recorded from patient’s clinical charts. Primary tumor features included tumor size, differentiation, Lauren histologic type, T stage, lymph node metastasis, lympho vascular invasion, and perineural invasion. The median tumor size was taken as the standard (median tumor size=4.8 cm), and the study defined tumors more than 5 cm in size as large tumors. The T stage was classified according to the American Joint Committee on Cancer Staging (AJCC), seventh edition (12).

### ^18^F-FDG-PET/CT imaging

Before ^18^F-FDG PET/CT scanning, all patients fasted for at least 6 hours, and blood glucose levels were less than 180 mg/dl. Scanning was initiated one hour after intravenous administeration of 7.4 MBq/Kg ^18^F-FDG. Images were obtained using a Gemini TF PET/CT scanner (Phillips Medical Systems, Cleveland, OH). Low-dose CT scan was obtained for attenuation correction, and the PET scan was followed at 1 min per bed position. PET images were reconstructed using 3D RAMLA (Row Action Maximum Likelihood Algorithm) with the CT data sets for attenuation correction.

A region of interest (ROI) was placed in the primary lesion, including the highest uptake area, and maximum standardized uptake values (SUV_max_) in the ROI were calculated.

### Immunohistochemistry

Paraffin blocks with representative areas of the tumors were cut into 4 µm thick tissue sections. Endogenous activity was quenched by incubation with 3% hydrogen peroxide for 30 minutes after deparaffinization and hydration. Antigen retrieval was subsequently carried out. The primary antibody used in this investigation was C-erbB2 (1:600; polyclonal, DAKO). Diaminobenzidine was used as a chromogen, and the slides were counterstained with hematoxylin.

**Figure 1:**
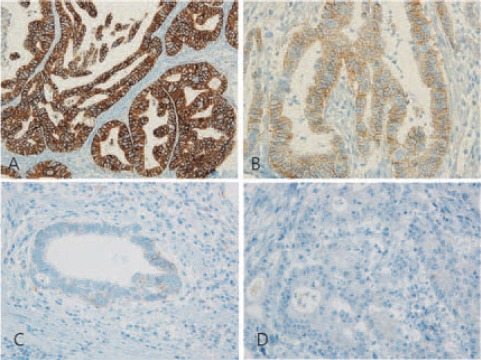
Immunohistochemical results for HER2 in gastric carcinoma. HER2 scores 3+ (A), 2+ (B), 1+ (C), and 0 (D).

All sections were evaluated by a pathologist who was blinded to the clinical characteristics of the patients. The pathologist scored slides as 0, 1+, 2+, or 3+ according to the DAKO manual for interpretation of HER2 staining (Figure 1). In this study, as in breast carcinoma, intensity scores of 2 and 3 were assumed to be positive for overexpression.

### Statistical analysis

Statistical analysis was performed with the Statistical Package for Social Sciences (SPSS) version 17.0. The correlations between levels of SUV_max_ and clinicopathologic factors, including HER2 overexpression, were evaluated using a *t*-test and one way ANOVA. The correlations between HER2 overexpression and clinicopathologic factors were evaluated using a chi-square test. Multiple linear regression analysis was performed to select independent clinicopathologic factors associated with SUV_max_. To identify an optimal cutoff value of SUV_max_ for the prediction of HER2 overexpression, receiver-operating characteristic (ROC) analysis was performed. Significance was considered as a *P*-value of less than 0.05.

## Results

### Patient characteristics

The patients included 80 (73.4%) males and 29 (26.6%) females with a mean age at the time of surgery of 63 years (age range, 34–87 years); 94 (86.2%) cases with tubular adenocarcinoma, 12 (11.0%) cases with signet ring cell carcinoma, and 3 (2.8%) cases with mucinous carcinoma. The mean tumor size was 4.9 cm (range, 1.2 to 18.0 cm); 58 (53.2%) cases were differentiated and 51 (46.8%) cases were poorly differentiated; 31 (28.5%) tumors were T2, 38 (34.8%) were T3, and 40 (36.7%) were T4. Lymph node metastasis and distant metastasis were observed in 73.4% and 9.2%, respectively.

### Correlation of SUV_max_ with clinicopathologic factors

The mean SUV_max_ for all 109 patient was 4.5, ranging from 0 to 21.7. The correlations of the level of SUV_max_ with clinicopathologic factors are presented in Table 1. The levels of SUV_max_ were significantly higher in large tumors than in small [≥5 cm, <5 cm, (5.6±3.8 vs. 3.5±2.3), *P*=0.02]. The mean level of SUV_max_ was significantly lower in poorly differentiated carcinoma than in differentiated carcinoma (2.8±2.3 vs. 5.9±3.1, *P*<0.001). The levels of SUV_max_ were significantly higher in intestinal type tumors than in diffuse type tumors (5.8±3.1 vs. 2.8±2.4, *P*=0.04). No association was found between SUV_max_ and T stage, lymph node metastasis, distant metastasis, lymph-vascular invasion, or perineural invasion.

**Table 1:** SUV_max_ and overexpression of HER2 in gastric carcinoma and association with clinicopathologic factors.

Category	SUV_max_		HER2 overexpression (%)		
	(mean±SD*)	*P*	0,1	2,3	*P*
Tumor size		0.02			0.53
<5 cm	3.5±2.3		46 (55.4)	14 (53.8)	
≥5 cm	5.6±3.8		37 (44.6)	12 (46.2)	
Differentiation		<0.001			0.22
Differentiated	5.9±3.1		42 (50.6)	16 (61.5)	
Poorly differentiated	2.8±2.3		41 (49.4)	10 (38.5)	
Histologic type by Lauren		0.04			0.71
Intestinal	5.8±3.1		45 (75.0)	15 (25.0)	
Diffuse	2.8±2.4		36 (76.6)	11 (23.4)	
Mixed	3.5±0.7		2 (100.0)	0 (0.0)	
T stage		0.70			0.61
T2	3.4±3.6		23 (27.7)	8 (30.8)	
T3	4.5±3.6		31 (37.3)	7 (26.9)	
T4	5.3±3.5		29 (34.9)	11 (42.3)	
LN metastasis		0.10			0.57
Absent	3.3±3.8		22 (26.5)	7 (26.9)	
Present	4.9±4.5		61 (73.5)	19 (73.1)	
Distant metastasis		0.84			0.44
Absent	4.5±4.3		76 (91.6)	23 (88.5)	
Present	4.2±3.2		7 (8.4)	3 (11.5)	
Lymphovascular invasion		0.07			0.14
Absent	3.2±3.7		21 (25.3)	10 (38.5)	
Present	5.0±4.5		62 (74.7)	16 (61.5)	
Perineural invasion		0.49			0.43
Absent	4.8±4.1		42 (50.6)	12 (46.2)	
Present	4.2±4.0		41 (49.4)	14 (53.8)	
HER2 overexpression					
Negative	3.9±2.1	0.02			
Positive	6.3±3.7				

* Standard deviation

### Correlation of HER2 overexpression with SUV_max_ and clinicopathologic factors

**Figure 2:**
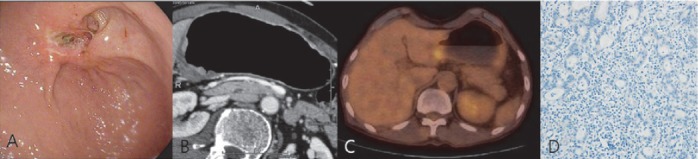
A case of negative HER2 immunostaining of advanced gastric carcinoma (Moderately differentiated tubular adenocarcinoma, pT4aN1, tumor size: 3×2.5cm). Upper gastrointestinal endoscopy view (A) and enhanced CT (B) showed ulcerative mass in the lesser curvature of gastric antrum. ^18^F- FDG PET-CT image (C) revealed focal increased FDG uptake in gastric antrum (SUV_max_ = 3.5). C-erbB2 immunostaining (D) showed negative result.

**Figure 3:**
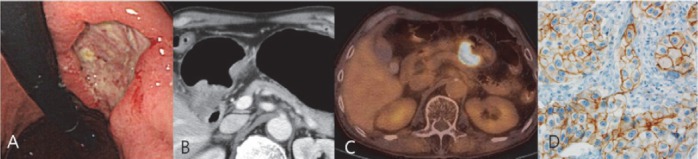
A case of 3+ HER2 immunostaining of advanced gastric carcinoma (Moderately differentiated tubular adenocarcinoma, pT4aN1, tumor size: 4.2×4cm). Upper gastrointestinal endoscopy view (A) and enhanced CT (B) showed ulcer fungating mass in the posterior wall of gastric antrum. ^18^F-FDG PET-CT image (C) revealed marked increased FDG uptake in gastric antrum (SUV_max_ = 19.7). HER2 immunostaining (D) showed strong complete membrane staining.

Immunohistochemically, 26 patients (23.8%) showed positivity for overexpression of HER2. The correlations of HER2 overexpression with clinicopathologic factors are presented in Table 1. The mean level of SUV_max_ in the tumor groups with scores 0 and 1+ was 3.9±2.1 and in the tumor groups with a score 2+ and 3+ was 6.3±3.7. The difference in SUV_max_ between the two groups was significant (*P*=0.02) (Figure 2, 3).

**Figure 4:**
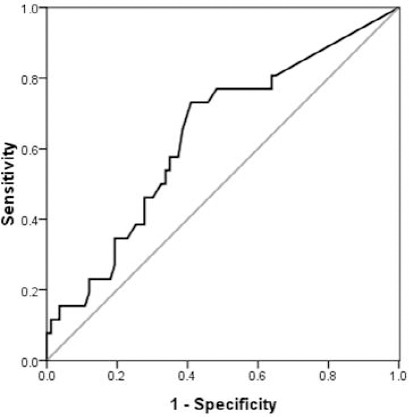
Receiver-operator characteristics (ROC) curve of SUV_max_ for predicting HER2 overexpression. The area under the ROC of SUV_max_ was 0.637. The sensitivity and specificity of dichotomized SUV_max_ (at cutoff value 3.5) were 76.9% and 60.2%, respectively.

A ROC curve of the SUV_max_ was plotted in order to predict HER2 overexpression. An area under the curve of 0.637 (*P*=0.036; 0.51<95% C.I.<0.75) was obtained (Figure 4). ROC curve demonstrated a SUV_max_ of 3.5 to be the optimal cutoff for predicting HER2 overexpression. The sensitivity of SUV_max_ for HER2 overexpression was 76.9% and its specificity was 60.2%. No association was found with HER2 overexpression and tumor size, T stage, lymph node metastasis, distant metastasis, lymphovascular invasion, and perineural invasion.

### Associations of SUV_max_ with clinicopathologic factors by multiple linear regression analysis

HER2 overexpression, larger tumor size, and differentiation (*P*=0.022, *P*=0.002, *P*<0.001) independently influenced the status of SUV_max_ in a multiple linear regression analysis (Table 2). The correlation coefficient (R^2^) of parameters with SUV_max_ was 0.212.

**Table 2:** Associations of SUV_max_ with clinicopathologic factors by multiple linear regression analysis

Variables	β^a^±SE^b^	Adjusted R^2^	*P* value
**Tumor size**	0.272±0.803	0.212	0.002
**Differentiation**	-0.498±1.118		<0.001
**Histologic type by Lauren**	0.203±0.746		0.094
**HER2 overexpression**	0.199±0.932		0.022

a Regression coefficient

b Standard errors

## Discussion

We evaluated the clinicopathologic characteristics and level of SUV_max_ of 109 cases with surgically resected AGCs. Our key findings include following: 1) The mean level of SUV_max_ is significantly higher in the group with HER2 overexpression; 2) The levels of SUV_max_ are significantly associated with tumor size, differentiation, and status of Lauren histologic type; 3) HER2 overexpression, tumor size, and differentiation are an independent significant indicator of the level of SUV_max_ in AGC.

SUV, a quantitative parameter commonly used in ^18^F-FDG PET/CT imaging, can provide information on aggressive tumor behavior. Previous studies have shown that the parameters for aggressiveness of cancer cells were strongly correlated with high levels of SUV_max_ (13).

In our study, the mean SUV_max_ level was significantly higher in intestinal type gastric carcinoma than in the diffuse type. We also found that the mean SUV_max_ level was significantly lower in poorly differentiated tumors than in differentiated tumors. Cancer cells over express glucose transporter-1 (GLUT-1), which is located in the cell membrane and facilitates the active uptake of ^18^F-FDG (14). Diffuse type gastric carcinomas, including signet ring cell carcinomas, display a consistently low detectability by ^18^F-FDG PET/CT (15, 16). This result is due to higher expression of GLUT-1 on the cell membrane of intestinal type gastric carcinoma (14, 17). Other factors influencing the low FDG uptake in diffuse type gastric carcinoma are the low tumor cell density and rich stroma (18). In addition to the Lauren classification, tumor size of gastric carcinomas was significantly associated with the level of SUV_max_ (16, 19, 20). FDG uptake is influenced by the number of metabolically active tumor cells (14). The larger the tumor size, the higher SUV is expected. Our results coincide with the findings of these previous studies.

Several studies also showed that a high SUV_max_ level was associated with lymph node metastasis. Oh *et al* (19) and Mochiki *et al* (20) reported that lymph node metastasis is associated with the level of SUV_max_. In the present study, no association was found with the level of SUV_max_ and lymph node metastasis. Although not statistically significant (*P*=0.07), the mean SUV_max_ level was higher in tumors with lymphovascular invasion than in tumors without lymphovascular invasion.

The HER2 gene has been recognized as a key regulator in the development of different type of tumors (21). Upon ligand binding, the receptors dimerize, are phosphorylated, and transduce intracellular signals that regulate cell prolifration, differentiation, and survival (21). In cancer formation, HER2 acts as an oncogene that regulates the proliferation, invasion, and apoptosis of cancer cells (22). Overexpression of HER2 protein was first described in gastric cancer in 1986 (23) and has since been correlated with poor outcome and a more aggressive disease (21, 22). Moreover, HER2 expression has become an increasingly important target for gastric carcinoma therapy (10). Several studies reported that the expression rate of HER2 varied from 4.4% to 53.4%. It was 23.8% in our study. This variability in the rate of HER2 expression is thought to arise from the use of non-standardized assays and different scoring criteria.

Many studies have demonstrated either an association or no association between HER2 overexpression and other factors including T stage, tumor grade, lymph node metastasis, and presence of metastasis (24). Hence, a definitive conclusion cannot be drawn from the literature regarding the association of HER2 overexpression with clinicopathologic markers. The only positive association of HER2 overexpression was with intestinal type gastric carcinoma (24, 25). In the present study, HER2 overexpression showed no association with tumor size, differentiation, Lauren histologic type, T stage, lymph node metastasis, distant metastasis, lymphovascular invasion, or perineural invasion. Standardization of HER2 testing procedures and interpretation is an essential step to ensure accurate and reproducible results.

In our study, the mean SUV_max_ level was significantly higher in cases of HER2 overexpression (score 2+ or 3+), and multivariate analysis showed that HER2 overexpression was independently influenced by the level of SUV in AGC. Therefore, the SUV_max_ of AGC might be a predictive factor for HER2 overexpression. This is supported by the result of the ROC curve, and the cutoff value of SUV_max_ was 3.5 (sensitivity 76.9%, specificity 60.2%). In contrast, Han *et al* (16) reported no correlation of the level of SUV_max_ with HER2 expression. The explanation for this difference is probably the application of different scoring criteria and the different range of patients. In the study by Han *et al*, HER2 positivity was defined as expression over 10%, excluding intensity. However, in our study, the patients were limited to those with AGC and the tumor groups were categorized into scores of 0 or 1+ versus scores of 2+ or 3+, taking into account both expression area and intensity. Further investigation is needed to characterize the mechanism underlying the association of high SUV_max_ with HER2 overexpression.

In conclusion, our results showed that a high SUV_max_ level is associated with HER2 overexpression, large tumor size, intestinal histologic type, and tumor differentiation in AGC. It might be possible to predict the biological characteristics of AGC using ^18^F-FDG PET/CT, but further studies are required to determine the clinical significance of the SUV level and its usefulness as biomarker of HER2 expression.
